# Selective Binding of Hardness Ions by Humic Sorbents for Prevention of Carbonate Scaling in Reverse Osmosis Systems

**DOI:** 10.3390/molecules31101677

**Published:** 2026-05-15

**Authors:** Alma Khassenovna Zhakina, Almat Maulenuly Zhakin, Yevgeniy Petrovich Vassilets, Oxana Vasilievna Arnt, Zainulla Muldakhmetov

**Affiliations:** 1 LLP “Institute of Organic Synthesis and Coal Chemistry of the Republic of Kazakhstan”, Karaganda 100008, Kazakhstan; zhakin-almat@mail.ru (A.M.Z.); vassilets88@mail.ru (Y.P.V.); oxana230590@mail.ru (O.V.A.); iosu.rk@mail.ru (Z.M.); 2 Department of Chemical Technology and Ecology, Faculty of Metallurgy and Mechanical Engineering, Karaganda Industrial University, Temirtau 101400, Kazakhstan

**Keywords:** humic acids, hardness ions, complexation, carbonate scaling, water–chemical stabilization, reverse osmosis, membrane processes, water treatment

## Abstract

This article examines the scientific basis for using humic acids for the chemical stabilization of mineralized water prior to reverse osmosis. The need to develop alternative approaches to water pretreatment is due to the limited effectiveness of traditional antiscale reagents at high mineralization, as well as their potential environmental risks and the likelihood of secondary contamination of water systems. The article focuses on the mechanisms of interaction between humic acids and Ca^2+^ and Mg^2+^ hardness ions, which are mediated by complexation with carboxyl and phenolic functional groups. It is demonstrated that humic stabilization differs from classical softening and demineralization in that it is aimed not at the complete removal of dissolved salts, but at reducing the activity of ions involved in the formation of carbonate deposits. The potential advantages of this approach for reducing the scale-forming potential of water, improving the stability of reverse osmosis membranes, and extending inter-flushing intervals are discussed. The technological limitations associated with residual organic load, possible membrane fouling, the need to control total organic carbon, color and stability of the filter medium, as well as a pilot test of the proposed approach are considered.

## 1. Introduction

**Figure 1:**
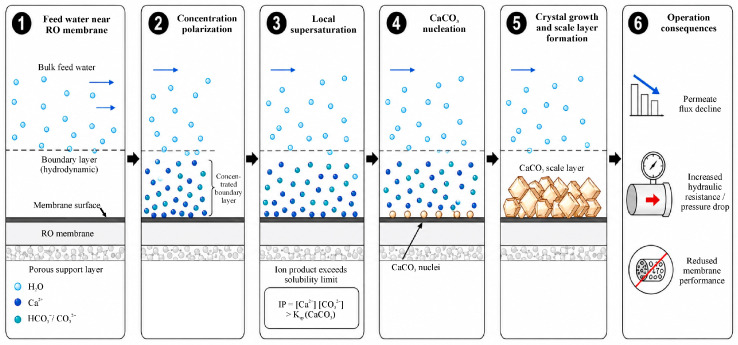
Literature-based schematic representation of CaCO_3_ scaling development on RO membranes.

In the 21st century, the problem of water resource scarcity is becoming increasingly important due to increased water consumption, population growth, and the persistence of unsustainable water use and management practices [1,2,3]. Given the growing scarcity of freshwater resources and increasingly stringent requirements for the operational reliability of water treatment systems, managing mineral scaling processes has become particularly important. Scale formation is a complex physicochemical crystallization process in which a solid phase is released from multicomponent supersaturated solutions. A generalized diagram of this process is presented in Figure 1, based on data from [4,5].

An analysis of literature data [6,7,8,9] shows that the formation of mineral deposits in membrane units and heat exchange equipment is a consequence of the combined action of physicochemical factors that determine the imbalance in the aqueous system. Among the most significant factors are supersaturation of the solution with poorly soluble salts, increased thermodynamic activity of hardness ions, changes in alkalinity, temperature, and hydrodynamic conditions, as well as a shift in the carbonate–calcium equilibrium. This creates conditions for the nucleation of a crystalline phase, particle growth, and their subsequent attachment to heat exchange surfaces or membranes. A summary of the research results indicates that carbonate, sulfate, and mixed mineral deposits not only reduce the efficiency of mass and heat transfer, but are also one of the main causes of increased hydraulic resistance, increased operating costs, and a shortened equipment service life.

An analysis of sources [10,11,12] allows us to conclude that scale formation in membrane and heat exchange systems is determined by the combined influence of water-chemistry and operational factors: the concentration of hardness ions, ionic composition, temperature, degree of concentration, hydrodynamic regime, and the state of the working surfaces. In reverse osmosis systems, this leads to local supersaturation of the solution in the boundary layer at the membrane surface, a decrease in their permeability, an increase in hydraulic resistance, an increase in specific energy consumption, and accelerated degradation of membrane elements. In heat exchange equipment, the formation of deposits is accompanied by a decrease in heat transfer efficiency, an increase in thermal resistance, and an increase in operating costs. A summary of literary data [13,14,15] also shows that existing methods for predicting and inhibiting scale formation remain limited, since their effectiveness significantly depends on changing water-chemistry, temperature, and hydrodynamic operating conditions.

Traditional water softening and demineralization technologies [16], including ion exchange, electrodialysis [17], electrodeionization [18], membrane separation, and capacitive deionization [19], are technologically developed and widely used approaches to reducing the mineralization of aqueous media. However, a summary of the literature data shows that the effectiveness of these methods largely depends on the initial ionic composition of the water, the level of mineralization, the operating parameters of the process, and the quality requirements for the purified water. When treating highly mineralized water, these technologies may be accompanied by increased energy and reagent costs, the formation of concentrates or regeneration solutions, and the need for an additional stage of water–chemical stabilization.

It should be noted that most traditional demineralization methods are focused primarily on reducing the total salinity or removing specific groups of ions, whereas the tendency of water to form scale is determined not only by the total salinity, but also by the thermodynamic activity of individual ions, their ratio, alkalinity, temperature, and mass transfer conditions in the process system [16,17,18,19]. Therefore, reducing the total salinity does not always ensure a proportional reduction in the risk of carbonate, sulfate, and mixed mineral deposits, especially under variable operating conditions.

Against this backdrop, a promising direction is the development of reagents and materials capable of not only reducing the concentration of individual ions but also regulating the nucleation, growth, and aggregation of mineral phases. In particular, humic polymer complexes [20] are considered more environmentally acceptable functional systems for binding metal ions and stabilizing mineralized waters due to the presence of carboxyl, phenolic, and other oxygen-containing groups. This approach shifts the emphasis from solely deep demineralization to selective management of water–chemical equilibrium and reducing the tendency of aquatic systems to form poorly soluble deposits.

A promising area of water–chemical stabilization is the use of humic acids as polyfunctional natural reagents capable of influencing the ionic equilibrium of aquatic systems and mineral formation processes. A summary of the literature data shows that the complexing capacity of humic acids is determined by the presence of carboxyl, phenolic, and other oxygen-containing functional groups involved in interactions with alkaline earth metal cations [21,22]. By binding Ca^2+^ and Mg^2+^ ions, humic acids can reduce their effective thermodynamic activity in solution, which reduces the likelihood of supersaturation with respect to poorly soluble carbonate phases [22].

Unlike traditional demineralization methods, which rely primarily on removing salts from water, the use of humic acids is aimed at regulating the processes of nucleation, growth, and aggregation of mineral phases without necessarily reducing the overall mineralization of the aquatic environment. Analysis of literature data indicates that humic acids are capable of inhibiting the growth of CaCO_3_ crystals, allowing them to be considered potential natural inhibitors of carbonate scale formation [23]. Thus, the use of humic acids is of interest not only as a method for binding hardness ions but also as an approach to selectively managing the carbonate–calcium balance and reducing the tendency of aquatic systems to form poorly soluble deposits.

The use of humic acids can be considered a promising approach to preliminary water–chemical stabilization prior to membrane processes, including reverse osmosis and nanofiltration. A review of the literature shows that the effectiveness of membrane technologies is largely determined not only by the selectivity and permeability of the membrane material, but also by the composition of the feedwater, the presence of natural organic matter, the solution’s tendency to mineralization, and the conditions under which fouling layers form on the membrane surface [24,25]. Therefore, pretreatment aimed at regulating organomineral interactions and reducing the activity of hardness ions is an important factor in increasing the resistance of membrane systems to fouling and scale formation.

An analysis of modern research indicates that reverse osmosis and nanofiltration processes should be considered not only as methods of deep separation and demineralization, but also as elements of complex process flowsheets sensitive to the composition of pre-treated water [24,25,26,27,28,29]. The presence of humic acids in aqueous systems can manifest itself in two ways: on the one hand, they can participate in the formation of organic membrane fouling, and on the other, due to their complexing and dispersing properties, they can influence the behavior of hardness ions and the crystallization processes of mineral phases. Therefore, of scientific interest is not the simple presence of humic acids in water, but the controlled use of humic acids as functional pre-treatment reagents, ensuring the stabilization of the aqueous medium prior to membrane separation.

From an anti-scale perspective, the use of humic acids may be associated with a reduction in the effective activity of Ca^2+^ and Mg^2+^ ions, altering the conditions for nucleation and growth of mineral phases, and limiting the attachment of crystalline particles to the membrane surface. This approach differs from traditional demineralization in that it is aimed not so much at the complete removal of salts as at managing their reactivity and tendency to form poorly soluble compounds. This is particularly important for reverse osmosis and nanofiltration systems, where localized salt concentration in the boundary layer near the membrane surface can significantly increase the risk of carbonate and mixed scale formation [24,25].

Modern trends in the development of membrane technologies also demonstrate a transition from the use of isolated separation processes to integrated water treatment schemes that include preliminary regulation of water composition, biochemical and physicochemical treatment stages, and the development of more stable membrane materials [24,25,28,29]. In this context, humic acids can act as natural polyfunctional reagents capable of binding Ca^2+^ and Mg^2+^ ions, changing the conditions for nucleation and growth of mineral phases, and reducing the likelihood of poorly soluble compounds attaching to the membrane surface. This approach differs from traditional demineralization, since it is aimed not at necessarily reducing total mineralization but at controlling the reactivity of ions and stabilizing the water system before membrane separation. Consequently, the prospects of this approach are determined not only by the ability of humic acids to bind hardness ions but also by the possibility of their controlled use in integrated water pretreatment schemes [24,25,26,27,28,29].

In contrast to traditional approaches focused on the removal of dissolved salts, this paper examines the concept of water–chemical stabilization based on managing the thermodynamic activity of hardness ions and carbonate equilibrium processes. It proposes considering humic acids not only as natural sorbents but also as functional reagents that selectively bind Ca^2+^ ions and modify the crystallization conditions of CaCO_3_. Additionally, a simplified modeling approach is proposed for comparatively assessing the susceptibility of water to carbonate scale formation under humic stabilization conditions.

## 2. Discussion

### 2.1. Reverse Osmosis in Water Treatment: Scale Formation and Operational Limitations

The formation of mineral scale is the result of the combined effects of water chemistry, temperature, pH, supersaturation, and hydrodynamic conditions. The most significant role in this process is played by the calcium-carbonate and carbon dioxide equilibria, as increasing temperature and changing pH promote the conversion of hydrocarbonates to carbonates and the subsequent formation of poorly soluble calcium and magnesium compounds. In addition to carbonates, scale may also contain sulfates, silicates, hydroxides, and metal oxides, complicating their prevention and removal [30].

In reverse osmosis systems, these processes are particularly intense due to the concentration of salts in the membrane wall layer and in the concentrate stream. This leads to an increase in the degree of supersaturation of the solution and creates conditions for the crystallization of mineral phases on the membrane surface. As a result, membrane permeability decreases, operating pressure increases, energy consumption increases, and the service life of membrane elements is shortened.

Consequently, scale formation is one of the key operational limitations of reverse osmosis water purification. Its prevention requires the use of effective pretreatment methods aimed at stabilizing the water’s salt composition, reducing the activity of hardness ions, and inhibiting the formation and growth of poorly soluble mineral phases.

Reverse osmosis systems are widely used for the preparation of drinking and industrial water; however, their operation is largely limited by processes of membrane fouling and scaling. Among the various degradation mechanisms, carbonate scaling caused by CaCO_3_ precipitation remains one of the main reasons for decreased membrane permeability, increased transmembrane pressure, and increased frequency of chemical cleaning. In this regard, preliminary stabilization of the water matrix before membrane processes is considered a key factor in improving the reliability and economic efficiency of reverse osmosis installations. In natural and technogenic waters, Ca^2+^ and Mg^2+^ ions play a significant role in the formation of carbonate hardness, and their concentration can vary significantly depending on the geochemical conditions of aquifers.

The high selectivity of semipermeable membranes enables significant reductions in total mineralization, making these systems popular in both drinking water production and industrial water treatment, including the energy and food sectors. Despite their technological advantages, the operation of reverse osmosis systems is subject to a number of limitations, the key one being membrane fouling. Depending on the nature of the fouling components, fouling can be classified as organic, colloidal, biological, or mineral. Among these mechanisms, mineral scaling, associated with the deposition of poorly soluble salts on the membrane surface, is particularly significant.

The most common form of mineral deposition in reverse osmosis systems is carbonate scaling, caused by the precipitation of CaCO_3_. This process occurs under conditions of localized supersaturation of the concentrated solution at the membrane surface, caused by concentration polarization. In the boundary layer, accumulation of Ca^2+^ ions and carbonate forms (HCO_3_^−^/CO_3_^2−^) occurs, which leads to an excess of the solubility product and initiates the processes of nucleation and crystal growth [8].

Carbonate scale formation is accompanied by a number of negative effects, including reduced membrane permeability, increased transmembrane pressure, increased specific energy consumption, and deterioration in permeate quality. Furthermore, deposit accumulation on the membrane surface alters hydrodynamic conditions and accelerates the development of other types of fouling, particularly organic and biological. Taken together, this reduces the service life of membrane elements and increases the frequency of chemical cleaning, which negatively impacts operating costs [5,31].

From a chemical thermodynamics perspective, the susceptibility of water to carbonate scaling is determined by its degree of supersaturation with CaCO_3_, which depends on the activity of Ca^2+^ ions and the carbonate system. Aqueous environment parameters such as pH, alkalinity, temperature, and ionic strength of the solution also play a significant role [32]. Increasing pH and removing CO_2_ during membrane filtration shifts the equilibrium of the carbonate system toward CO_3_^2−^ formation, which increases the likelihood of CaCO_3_ precipitation.

An additional factor in scale formation is concentration polarization, where dissolved salts accumulate in the boundary layer near the membrane surface. As a result, the local ion concentration can significantly exceed their content in the bulk solution, leading to supersaturation and the initiation of precipitation of poorly soluble compounds even with moderate source water mineralization. In natural and industrial waters, Ca^2+^ and Mg^2+^ ions play a significant role in the formation of carbonate hardness. Their concentrations are determined by the geochemical conditions of the aquifers and can vary significantly. Ca^2+^ ions, which have a higher tendency to form poorly soluble compounds, are the primary factor in the formation of carbonate deposits in membrane systems.

To prevent scale formation in reverse osmosis systems, various water pretreatment methods are used, including chemical softening, dosing of scale inhibitors (antiscalants), decarbonation, and pH regulation. However, each of these approaches has its limitations. Chemical softening, for example, is associated with the formation of scale and the need for its removal, the use of antiscalants requires precise composition and dosage, and changes in pH can lead to side effects, including equipment corrosion and changes in water properties. Therefore, increasing attention is being paid to approaches aimed not at removing salts, but at managing their thermodynamic activity and crystallization processes. Preliminary water–chemical stabilization is considered a promising approach for reducing the tendency of water to form scale without significantly altering its total ionic composition.

Thus, the limiting factors in the operation of reverse osmosis systems are directly related to carbonate scale formation, which is determined by both the composition of the feedwater and the operating conditions of the membranes. This necessitates the development and implementation of effective methods for pre-stabilizing the water matrix, focused on managing ionic equilibrium and preventing mineral deposit formation. In this regard, methods aimed not at removing salts but at controlling their thermodynamic activity and phase formation processes are of particular interest.

### 2.2. Humic Acids as Functional Reagents for Water–Chemical Stabilization: Conceptual Differences from Demineralization and Limitations of Application in Water Treatment

Traditional water treatment technologies, including ion exchange, reverse osmosis, nanofiltration, electrodialysis, and thermal demineralization, are primarily aimed at reducing the total salt content of water and removing dissolved minerals. Despite their high efficiency, their use can be associated with significant energy and reagent consumption, the formation of concentrated salt streams, and the risk of secondary contamination. Furthermore, reducing mineralization does not always completely prevent scale formation, as scale formation depends not only on the concentration of ions but also on their activity, chemical form, and crystallization conditions.

**Figure 2:**
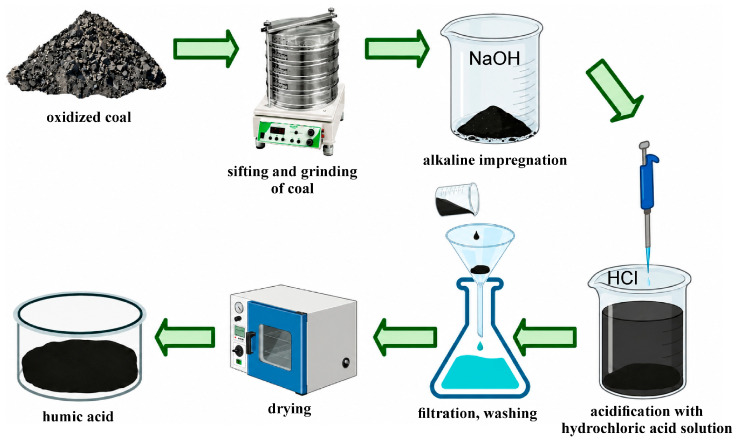
The main stages of the technological process for obtaining humic acids from oxidized coal.

Unlike demineralization, water–chemical stabilization is aimed not so much at removing dissolved components as at regulating the ionic equilibrium of the aquatic system. This approach is based on changing the conditions under which Ca^2+^, Mg^2+^, CO_3_^2−^, and HCO_3_^−^ ions transform into poorly soluble mineral phases. The key factor here is not the absolute concentration of the ions, but their thermodynamic activity, which determines the likelihood of the formation of poorly soluble compounds, primarily CaCO_3_. From a chemical thermodynamics perspective, CaCO_3_ precipitation is possible when the product of the activities of Ca^2+^ and CO_3_^2−^ ions reaches or exceeds the solubility product. This process depends on pH, alkalinity, temperature, ionic strength of the solution, the ratio of carbonate forms, and the presence of organic and inorganic complexing agents. In the context of water treatment, humic acids are of the greatest scientific and applied interest as a natural polyfunctional fraction of humic substances, possessing a developed system of acid–base, complexing and sorption-active centers [33,34,35,36,37,38,39]. Their structural organization is determined by the presence of carboxyl, phenolic, hydroxyl, carbonyl and other oxygen-containing functional groups capable of participating in complex formation, ion exchange, electrostatic interaction and adsorption binding of metal cations [40,41,42,43,44,45,46,47,48]. Therefore, within the framework of the concept of water–chemical stabilization, humic acids should be considered not only as natural organic substances or sorbents, but as functional reagents capable of changing the distribution of ionic forms in aqueous systems and influencing the conditions of formation of poorly soluble mineral phases. Figure 2 shows the process flow diagram for obtaining humic acids from oxidized coal.

An analysis of the literature [49,50,51,52,53,54,55] shows that the use of humic acids in water treatment technologies has been studied for several decades; however, information on their practical use remains fragmentary and ambiguous. This is due to the fact that most studies have traditionally focused on the sorption of heavy metal ions, organic micropollutants, and radionuclides, whereas the role of humic acids in regulating water hardness, calcium-carbonate equilibrium, and antiscale stabilization processes has been studied considerably less. Humic acids have been shown to be capable of binding Pb^2+^, Cu^2+^, Zn^2+^, and other cations through interaction with carboxyl and phenolic groups of the macromolecular structure. However, direct transfer of these patterns to the Ca^2+^/Mg^2+^–HCO_3_^−^CO_3_^2−^ systems requires caution, since scale formation processes are determined not only by the total sorption capacity, but also by the activity of free ions, the degree of supersaturation of the solution, and the kinetics of crystallization of mineral phases.

**Figure 3:**
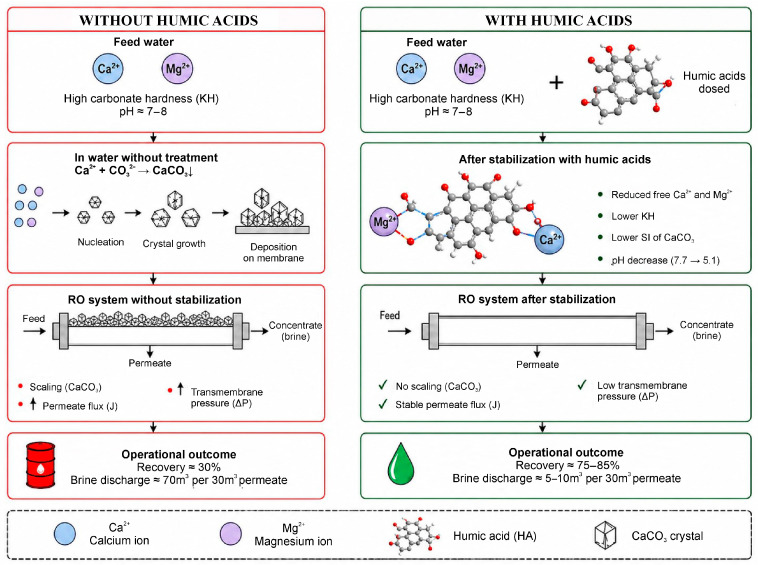
Schematic illustration mechanism of Ca^2+^ binding by humic acids and its effect carbonate scaling and operational parameters of reverse osmosis systems.

The mechanism of action of humic acids on hardness ions differs fundamentally from traditional water softening, which removes Ca^2+^ and Mg^2+^ from the aqueous medium. In this case, the key role is played not by the removal of hardness ions, but by changes in their chemical state and reactivity. According to literature, humic acids are capable of interacting primarily with Ca^2+^ ions to form labile soluble or colloidal-associated complexes, which leads to a decrease in the activity of free cations in solution. This binding limits the participation of Ca^2+^ in the formation of poorly soluble carbonate phases, reduces the system’s tendency to scale formation, and thereby contributes to increased operational stability of membrane systems, including reverse osmosis systems. A schematic illustration of the mechanism of Ca^2+^ binding by humic acids, its effect on carbonate deposition, and the operating parameters of reverse osmosis systems is shown in Figure 3.

In this case, Ca^2+^ and Mg^2+^ are not necessarily removed from the solution, but the proportion of their free, thermodynamically active forms is reduced. From a practical standpoint, this means that the total analytical concentration of hardness ions may change slightly, while their contribution to CaCO_3_ formation decreases. Therefore, it is inappropriate to evaluate humin-based methods solely by criteria applied to demineralization or reagent softening. Their effectiveness should be assessed through changes in Ca^2+^ activity, supersaturation indices, the equilibrium distribution of carbonate forms, and the tendency of water to form CaCO_3_.

The most pronounced complexing effect of humic acids is manifested in the neutral and slightly alkaline pH range, where a significant portion of the carboxyl groups are in ionized form. Under these conditions, the ability of humic macromolecules to coordinate Ca^2+^ and Mg^2+^ cations increases, which can lead to a shift in the calcium-carbonate equilibrium and a reduced likelihood of reaching a critical degree of supersaturation with CaCO_3_. However, this effect is not universal. At low pH values, the functional groups of humic acids are partially protonated, which reduces their complexing capacity. Conversely, at high ionic strengths, charge shielding, competition between cations for active sites, and changes in the conformational state of humic macromolecules are possible. Therefore, the results obtained on model solutions with low mineralization cannot always be directly extrapolated to natural, recycled, or industrial waters of complex composition.

A significant aspect of the antiscale action of humic acids is their influence not only on chemical equilibrium but also on the kinetics of phase formation. Complexation with Ca^2+^ reduces the concentration of free calcium involved in CaCO_3_ formation, but this is not the only inhibition mechanism. Humic acid macromolecules can adsorb on the surface of nascent CaCO_3_ particles, blocking active growth centers, altering the surface energy of the mineral phase, and disrupting the regular formation of the crystal lattice. As a result, nucleation, aggregation of primary particles, and crystal growth are slowed, and the size, morphology, and phase-morphological characteristics of the resulting carbonate precipitates are altered. This mechanism is particularly important for membrane processes, since operational problems in reverse osmosis are associated not only with the amount of precipitate formed but also with the nature of its formation on the membrane surface.

However, the use of humic acids in water treatment is associated with a number of limitations that must be considered when assessing their technological potential. First, the introduction of humic acids can increase the content of dissolved organic matter, water color, and oxidation indices. When administered uncontrolled, humic acids can act as an additional source of organic load, which is particularly important for membrane systems sensitive to organic contamination. Second, the effectiveness of humic acids depends significantly on their origin, oxidation state, molecular weight distribution, content of oxygen-containing functional groups, and pretreatment conditions. The lack of uniform standards for humic-based materials complicates the comparison of results from various studies and limits the scalability of laboratory data [43,48,49,50].

Thus, a critical analysis of the literature suggests that the potential of humic acids in water treatment is most fully realized not when considered as universal sorbents or demineralization substitutes, but when used as functional reagents for water–chemical stabilization. Their action is associated with a combination of interconnected mechanisms: a decrease in the activity of free Ca^2+^ and Mg^2+^ ions, changes in the calcium-carbonate equilibrium, a decrease in the degree of water supersaturation relative to CaCO_3_, and adsorption inhibition of the nucleation and crystal growth of poorly soluble mineral phases. The effectiveness of humic acids should be assessed not only by the reduction in the mass of deposits formed, but also by changes in the calcium species, supersaturation indices, crystallization kinetics, precipitate morphology, and the operational stability of process equipment. This approach is of particular interest for pre-treatment of water before membrane processes, including reverse osmosis, since it reduces the tendency of water to scale formation without significantly changing the total salinity, but requires strict control of dosage, the composition of humic reagents and the organic load at subsequent stages of purification.

### 2.3. Prospects for the Use of Humic Acids: Preliminary Water–Chemical Stabilization Before Reverse Osmosis

Traditional methods of protecting membranes from carbonate scaling include dosing of antiscalants, acidification of water, and partial softening. Despite their effectiveness, these approaches have a number of disadvantages associated with the need for precise dosing control, the risk of secondary contamination, and accumulation of chemical reagents in the concentrate. In addition, antiscalants do not eliminate the cause of scaling, but only inhibit crystal growth, which limits their effectiveness under fluctuations in the composition of feed water [56,57].

In this context, water–chemical stabilization using humic acids represents an alternative or auxiliary approach to reducing carbonate scaling tendency. Due to complexation with Ca^2+^ ions and modification of the equilibrium of the carbonate system, humic acids reduce the activity of Ca^2+^ and decrease the probability of exceeding the solubility product of CaCO_3_ [58]. Unlike antiscalants, the action of humic acids is based on redistribution of ionic forms in solution rather than on kinetic inhibition of crystallization. Table 1 shows the data from water analyses using modified humic acids at the “Institute of Organic Synthesis and Coal Chemistry of the Republic of Kazakhstan” (Karaganda, Kazakhstan).

A specific feature of humic water–chemical stabilization is that its effectiveness in suppressing scaling is determined not by a decrease in total mineralization of water, but by a reduction in carbonate hardness and calcium activity. This makes this approach particularly attractive for the pretreatment of waters with high mineralization, where the use of membrane demineralization at early stages is associated with high energy costs and the risk of intensive scaling [22,28].

**Figure 4:**
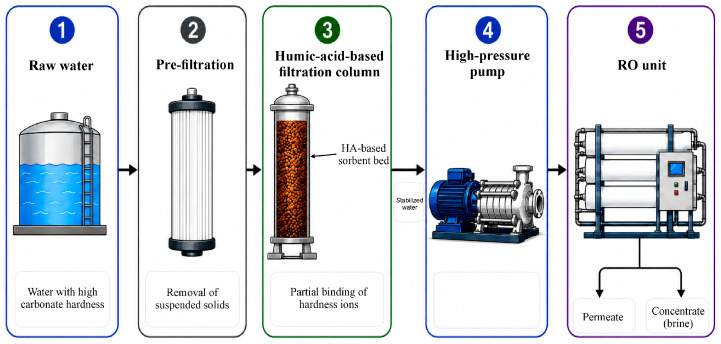
Proposed technological scheme of feed-water stabilization using a humic-acid-based filtration column prior to reverse osmosis.

From an engineering point of view, treatment based on modified humic acids can be integrated into water treatment schemes as a stage of preliminary water–chemical stabilization before reverse osmosis. This stage can be implemented, in particular, as a humic-acid-based filter column, which binds hardness ions and reduces the water’s tendency to form carbonate scale. Depending on the adopted process flow diagram, the process can be accompanied by subsequent removal of excess organic components by coagulation, adsorption, or ultrafiltration. Implementation of this approach reduces carbonate hardness, stabilizes the ionic composition of the feedwater while maintaining a controlled level of organic loading, reduces membrane fouling, and increases the intervals between flushes in reverse osmosis units. The proposed process flow diagram for feedwater stabilization using a humic-acid-based filter column prior to reverse osmosis is shown in Figure 4.

It should be noted that the use of humic acids as a pretreatment stage does not exclude the application of other protective measures, but rather can be considered as part of an integrated strategy. Specifically, humic-acid-based water–chemical stabilization can reduce the dosage of scale inhibitors, reduce the need for acidification, and increase the system’s resilience to fluctuations in feedwater composition. This integrated approach is consistent with modern trends in membrane technology development, which are focused on optimizing overall operating parameters rather than solely improving individual indicators.

**Figure 5:**
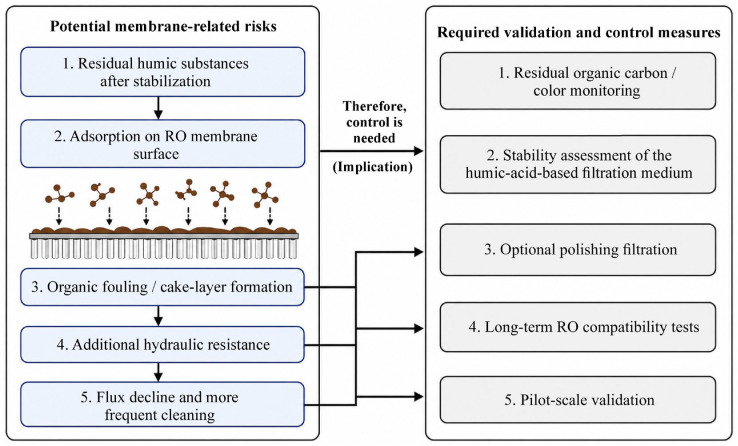
Potential membrane-related risks and validation needs for humic-acid-based stabilization prior to RO.

However, despite the potential effectiveness of humic water–chemical stabilization in reducing the activity of Ca^2+^ and Mg^2+^ ions, the use of humic materials before the reverse osmosis stage requires additional membrane risk assessment. Residual dissolved or colloidal humic acids, if insufficiently fixed in the filter medium, can adsorb on the membrane surface, contribute to the formation of an organic fouling layer, and increase hydraulic resistance. In the long term, this could lead to a reduction in the standardized flow and an increase in the frequency of chemical cleanings. Therefore, the proposed technology should not be considered a fully integrated industrial solution, but rather as a promising preliminary stabilization scheme requiring monitoring of residual organic carbon and color, assessment of the stability of the humic filter media, verification of the need for final filtration, as well as long-term compatibility testing with RO membranes and pilot validation. A summary of potential membrane risks and necessary control measures is presented in Figure 5.

Thus, humic-acid-based water–chemical stabilization represents a promising approach for reducing carbonate scale formation in reverse osmosis systems. The chemical mechanisms of complexation and modification of carbonate equilibrium underlying this approach allow humic acids to be considered functional reagents capable of increasing the operational stability of membrane processes without the need for complete water demineralization in the early stages of water treatment. It should be emphasized that this scheme is not a standalone alternative to traditional scale prevention methods, but should be considered as part of a hybrid technological strategy. Its effectiveness is primarily demonstrated in waters with elevated carbonate hardness, while in low-mineralization systems, the effect of humic acids on scale formation may be limited. Furthermore, the use of humic materials does not completely suppress carbonate deposition, but only reduces its intensity, necessitating additional membrane protection methods, including pH regulation, inhibitors, and periodic backwashing. Practical implementation of this scheme requires consideration of the saturation of the humic filter medium and the possible reduction in its effectiveness over time due to the accumulation of bound Ca^2+^ ions. This necessitates regeneration or replacement of the media, as well as monitoring the stability of its sorption properties.

### 2.4. Model Approach for Evaluating the Reduction of Carbonate Scaling Tendency Under Humic Water–Chemical Stabilization

To substantiate the applied potential of water–chemical stabilization based on humic acids prior to membrane processes, it is advisable to use simplified model approaches that allow linking changes in the ionic composition of water with the operational characteristics of reverse osmosis systems. Within review studies, such models do not claim to provide an exact description of thermodynamic equilibrium; however, they make it possible to demonstrate key trends associated with the suppression of carbonate scaling.

Since the formation of CaCO_3_ deposits is primarily determined by the activity of Ca^2+^ ions and carbonate species, the target parameter for modeling was chosen not as the total mineralization of water, but as the carbonate component of hardness. To assess the relative tendency of water toward carbonate scaling, a simplified calculated indicator of carbonate scaling tendency was introduced, proportional to the product of calcium concentration and carbonate hardness, SI:

(1) $$SI=C_{Ca}\cdot {KH}_{SI}$$

where C_Ca_ is the calcium concentration in mmol/L, and KH is the carbonate hardness expressed in degrees of hardness. This approach reflects the chemical nature of the scaling process, in which the decisive role is played by the availability of carbonate forms of calcium ions rather than by the total salt content of water.

To compare different conditions, a relative scaling tendency indicator was introduced:

(2) $$S=\frac{SI}{{SI}_{0}}$$

where SI_0_ corresponds to the initial water without conditioning. Values of S < 1 indicate a decrease in the potential tendency toward carbonate scale formation. The influence of carbonate scaling on the operational characteristics of membrane systems can be described through changes in normalized membrane permeability over time. Within the framework of a simplified approach, the decrease in normalized reverse osmosis flux is represented by an exponential dependence:

(3) $$\frac{J(t)}{J}=exp[-\left(\alpha_{0}+\alpha_{s}S\right)t]$$

where J(t) is the flux through the membrane at time t, J_0_ is the initial flux, α_0_ is the contribution of baseline degradation factors (concentration polarization, organic fouling), and α_s_ is the contribution associated with carbonate scaling.

**Figure 6:**
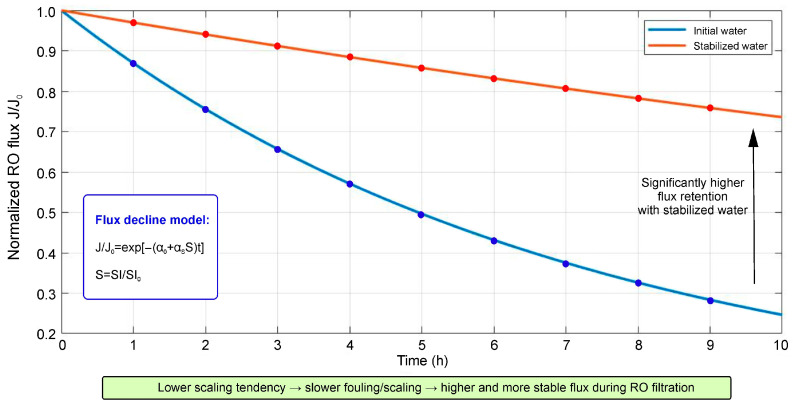
Predicted membrane flux decline after humic-acid-based stabilization.

Figure 6 presents model calculations showing that a decrease in carbonate hardness and calcium activity as a result of humic conditioning leads to a reduction in the value of parameter S and, consequently, to a decrease in the contribution of the scaling component to membrane flux degradation. At the same time, even under conditions of increased total mineralization of water, a slower decrease in normalized reverse osmosis flux is predicted due to suppression of CaCO_3_ scaling processes.

The proposed SI should not be considered as a replacement for classical saturation indices such as LSI or full chemical specification calculations, but as a simplified comparative parameter reflecting changes in calcium availability and the carbonate component of hardness. This approach is consistent with the general principles of assessing carbonate scale formation, according to which the probability of CaCO_3_ precipitation is determined by the degree of solution supersaturation, the activity of Ca^2+^ and CO_3_^2−^ ions, pH, alkalinity, temperature, and ionic strength of the solution [32,53,54,55]. For membrane systems, concentration polarization is of additional importance, enhancing local supersaturation at the membrane surface [4,5,31,56]. The effect of humic acids on this process is associated with Ca^2+^ binding, a decrease in the activity of free calcium, and inhibition of CaCO_3_ crystal growth [22,23,48,58]. It should be emphasized that the proposed model approach is illustrative in nature and is intended to demonstrate the fundamental possibility of using humic acids as a stage of preliminary water–chemical stabilization. For accurate quantitative prediction of the operational characteristics of membrane systems, it is necessary to take into account the full chemical specification of water, including pH, alkalinity, ionic strength, and the presence of inhibiting or catalytic components. Nevertheless, even in simplified form, the model highlights the key role of changes in ionic equilibrium and carbonate hardness when evaluating the effectiveness of water–chemical stabilization based on humic acids.

Thus, the use of model approaches focused on chemically justified indicators of scaling tendency makes it possible to substantiate the applied potential of humic acids in pretreatment schemes before reverse osmosis and serves as a link between fundamental chemical mechanisms and engineering aspects of membrane system operation. It should be noted that the proposed modeling approach is primarily applicable to waters with pronounced carbonate hardness, where changes in Ca^2+^ activity have a significant impact on the degree of CaCO_3_ supersaturation. In waters with low mineralization or where other pollution mechanisms (e.g., organic or colloidal) predominate, the applicability of this model is limited.

## 3. Conclusions

The analysis showed that humic acids can be considered natural multifunctional reagents for the water–chemical stabilization of mineralized waters prone to carbonate scale formation. Their action is not due to the direct removal of hardness ions, but rather to a change in their chemical state in the aqueous phase. The presence of carboxyl, phenolic, and other oxygen-containing functional groups ensures the binding of Ca^2+^ and Mg^2+^ ions to form soluble or colloidally associated complex forms, which reduces the activity of free hardness ions and limits their participation in the formation of poorly soluble carbonate phases. It was shown that the effectiveness of humic stabilization is determined not by a reduction in total mineralization but by changes in the activity of hardness ions and the conditions of carbonate equilibrium.

The fundamental difference between humic-acid-based water–chemical stabilization and classical demineralization is that the effectiveness of this treatment cannot be assessed solely by total mineralization or dry residue. Even if the content of the dissolved organic phase is maintained or increased, it is possible to reduce the scale-forming potential of water due to the redistribution of ionic forms, a decrease in the activity of Ca^2+^ and Mg^2+^, and stabilization of the carbonate–calcium equilibrium.

For reverse osmosis systems, humic acids should be considered not as a standalone, universal purification technology but as a potential component of the preliminary water–chemical stabilization stage. This approach can be aimed at reducing carbonate hardness, mitigating the risk of CaCO_3_ formation on the membrane surface, and extending the intervals between flushes. However, the use of humic reagents requires mandatory monitoring of the residual organic load, as excess dissolved or colloidal organic phase can increase organic fouling of the membranes and reduce the stability of reverse osmosis systems.

One of the main limitations of current research is the lack of standardization of humic acid composition, which depends on the origin of the raw materials, the isolation method, the molecular weight distribution, and the content of functional groups. Furthermore, the literature lacks comparable data on the stability of Ca^2+^/Mg^2+^ complexes with humic acids in real water matrices containing HCO_3_^−^, SO_4_^2−^, Cl^−^, Fe and Si compounds in the form of oxides, hydroxides, and silicates, as well as a natural organic matrix. This complicates quantitative prediction of the antiscale effect and the transfer of laboratory results to pilot and industrial conditions.

In further research, it would be advisable to employ experimentally validated methodological approaches, including determining the complexing capacity of humic acids with respect to Ca^2+^ and Mg^2+^ ions, assessing changes in CaCO_3_ saturation indices before and after treatment, dynamic testing in filter columns, and testing in laboratory or pilot RO units with controlled membrane flow, pressure drop, salt retention, and chemical flushing frequency. This integrated approach will allow for an objective assessment of the effectiveness of humic acids as pre-stabilizing reagents for water and the determination of process conditions under which their use does not lead to secondary organic fouling of membranes. The proposed approach should be considered as a component of hybrid water treatment schemes, requiring further experimental validation and optimization, taking into account the water composition and the operating parameters of the membrane systems.

## Figures and Tables

**Table 1:** Water analysis data before and after treatment with humic acids.

Parameter	Symbol	Units	Initial Water	Stabilized Water
Ca^2+^ ion concentration	Ca^2+^	mg/L	100	56
Mg^2+^ ion concentration	Mg^2+^	mg/L	49	36
Carbonate hardness	KH	°dH	4.0	0.2
pH	–	–	7.7	5.1

## Data Availability

No new data were created or analyzed in this study.
